# The influence of whispering gallery modes on the far field of ring lasers

**DOI:** 10.1038/srep16668

**Published:** 2015-11-17

**Authors:** Rolf Szedlak, Martin Holzbauer, Donald MacFarland, Tobias Zederbauer, Hermann Detz, Aaron Maxwell Andrews, Clemens Schwarzer, Werner Schrenk, Gottfried Strasser

**Affiliations:** 1 Institute of Solid State Electronics, TU Wien, Floragasse 7, 1040 Vienna, Austria; 2 Center for Micro- and Nanostructures, TU Wien, Floragasse 7, 1040 Vienna, Austria

## Abstract

We introduce ring lasers with continuous *π*-phase shifts in the second order distributed feedback grating. This configuration facilitates insights into the nature of the modal outcoupling in an optical cavity. The grating exploits the asymmetry of whispering gallery modes and induces a rotation of the far field pattern. We find that this rotation can be connected to the location of the mode relative to the grating. Furthermore, the direction of rotation depends on the radial order of the whispering gallery mode. This enables a distinct identification and characterization of the mode by simple analysis of the emission beam.

The first description of whispering gallery waves^1^ was conducted by Lord Rayleigh in the 19th century. He showed that sound waves in the St. Paul’s Cathedral in London exhibit more losses on the direct path than on the circular path along the gallery. This principle of sound waves can be applied to light waves^2^ and electrons^3^. Whispering gallery modes (WGMs) travel along circular cavities exploiting the total internal reflection. The cavity must contain an integer number of mode oscillations in order to fulfill the phase matching condition after one round trip. In the last two decades a lot of effort went into the development of proper techniques in order to experimentally map and visualize the shape of WGMs. With the utilization of near field probes^4^,^5^, photon scanning tunneling microscopy^6^ and thermocouple probes^7^ it was possible to confirm the theoretical predictions. Furthermore, WGMs were successfully used in slightly asymmetric circular cavities for rotation dependent far field patterns^8^ as well as unidirectional laser emission^9^.

In this paper we investigate the influence of WGMs on the far field of ring lasers with a modified distributed feedback (DFB) grating. Though this resonator and DFB design is demonstrated here for mid-infrared ring quantum cascade lasers (QCLs)^10^, it applies to other semiconductor or high refractive index lasers with a DFB grating for light outcoupling as well. The first QCL^11^ was demonstrated in 1994, more than 20 years after the theoretical foundation^12^ was established. Due to their customizable emission wavelengths currently ranging from 2.6 μm^13^ in the near-infrared to 441 μm^14^ in the terahertz spectral region, QCLs are popular tools for spectroscopic applications like chemical sensing of gases^15^ and liquids^16^. QCLs promoting WGMs were already realized in the form of disks^9^,^17^,^18^ exhibiting in-plane emission due to scattering processes, and rings^19^,^20^,^21^ with vertical emission through a second order DFB grating. This grating provides optical feedback and determines the outcoupling of the light^22^,^23^. It has been shown that modifications of the DFB grating, both for straight ridges^24^,^25^,^26^ and ring lasers^27^, strongly influence the device characteristics. Previously, we reported on ring QCLs with two abrupt *π*-phase shifts^28^ in the DFB grating. We successfully demonstrated that the controlled alteration of the phase provides a far field with a central intensity maximum. This is comparable to the effect in ridge type devices with one central grating phase shift^29^,^30^.

## Results

### Dual distributed feedback grating with continuous *π*-phase shifts

**Figure 1:**
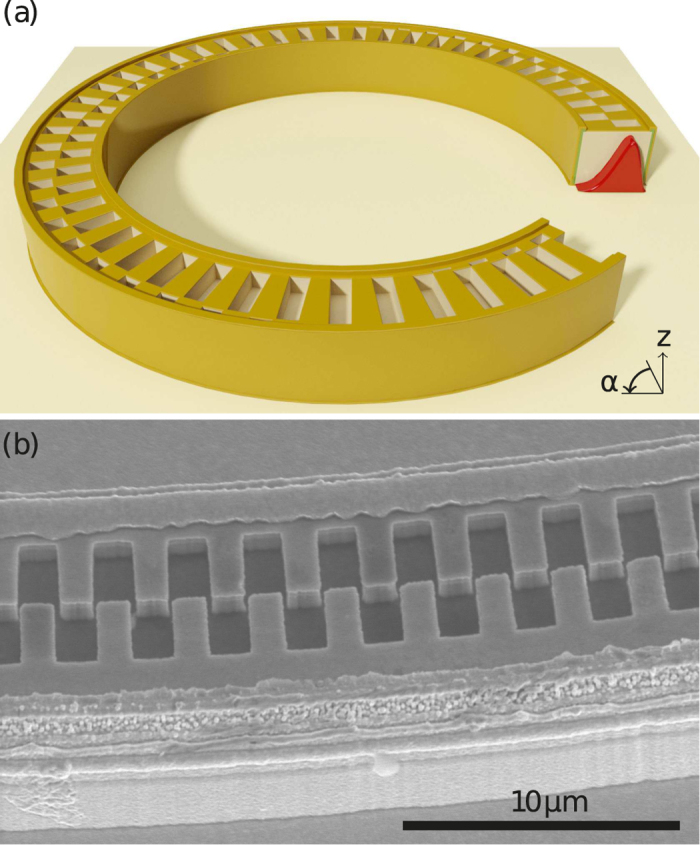
(**a**) Sketch of a ring laser with two continuous *π*-phase shifts in the DFB grating. The cross-section discloses how the WGM (red) is located within the waveguide. (**b**) Scanning electron microscopy image of the dual grating.

**Figure 2:**
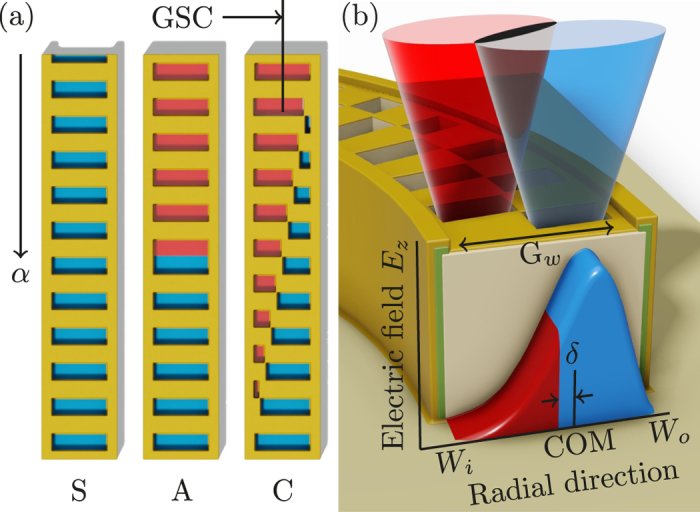
(**a**) Comparison between a standard (S), an abrupt *π*-shift (A) and a continuous *π*-shift (C) grating. For the latter the radius of the grating slit center (GSC) is marked. The red and blue grating slits symbolize the *π*-shifted and unshifted grating elements, respectively. (**b**) Dual grating above a WGM. The colored light cones couple out the corresponding parts of the WGM. The relative phase shift between the light cones induces destructive interference. Inner and outer waveguide radii are given by W_*i*_ and W_*o*_, respectively. G_*w*_ denotes the total grating slit width and *δ* is given by the distance between the GSC and the center of mass (COM) of the WGM.

**Figure 3:**
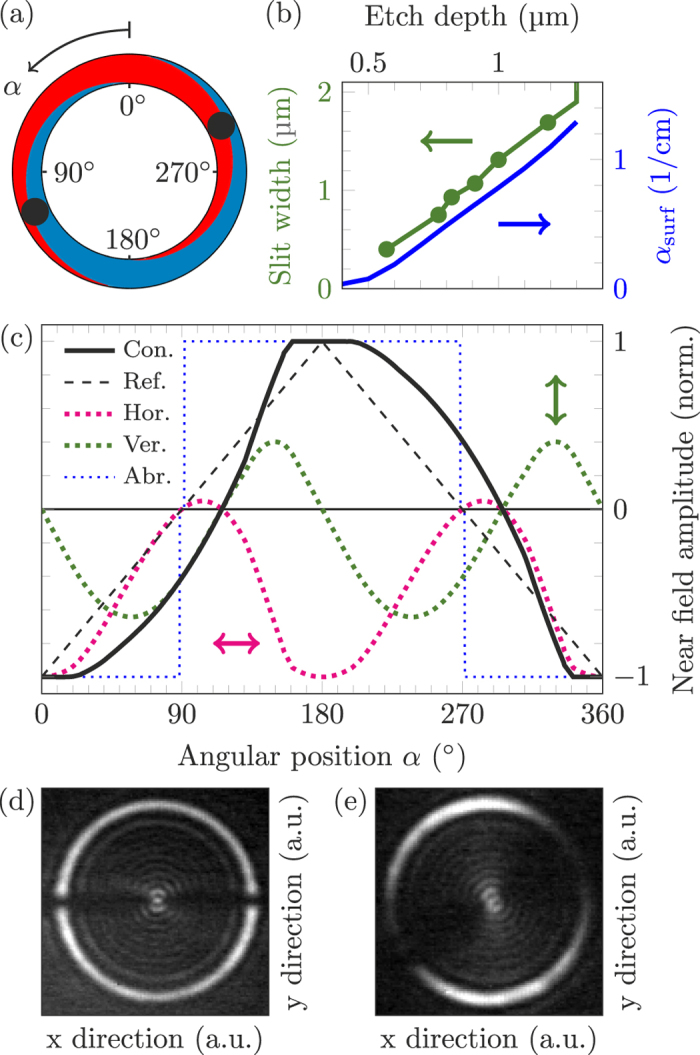
(**a**) Sketch of two continuous phase shifts. The black circles represent the locations of strongest light annihilation. (**b**) Slit width and calculated surface losses as a function of the etch depth. Small grating slits exhibit a reduced etch depth and therefore a weaker light emission. (**c**) Calculated electric near field amplitude *E*_NF_ of a continuous *π*-shift (solid black) and reference amplitude (dashed black) along the ring. The horizontal (dotted magenta) and vertical (dotted green) polarization components are given by the product of *E*_NF_ with a cosine and sine function, respectively. The faint kinks at 0° ± 19° and 180° ± 19° originate from the onset of the dual grating region. The dotted blue line represents the near field amplitude of an abrupt *π*-phase shift. Near field intensity pattern of an abrupt (**d**) and a continuous (**e**) *π*-phase shift ring QCL.

In contrast to these abrupt grating phase shifts, we introduce here a novel DFB grating design with two continuous *π*-phase shifts. A sketch of a ring laser with such a grating is given in Fig. 1(a). The phase shift consists of a dual grating configuration. The two grating vectors *G*_*B*_ = *G*_0_ and *G*_*R*_ = *G*_0_*e*^*iπ*^ are shifted by a phase value of *π* with respect to each other. A scanning electron microscopy image of this dual grating structure is given in Fig. 1(b). At the cross-section shown in Fig. 1(a), the inner part of the grating is phase shifted by *π* and the outer part is unshifted. The situation is reverse for the other side of the ring. The ratio between the shifted and unshifted grating elements is continuously varying along the ring. This means that the phase shift is not localized but spread over a large area of the ring. The continuous *π*-phase shift grating is compared to a standard and an abrupt *π*-shift grating in Fig. 2(a). Without loss of generality, we can define the blue grating slits as unshifted and the red slits as *π*-shifted. Figure 2(b) shows the electric field amplitude of the WGM below a grating element of a continuous phase shift grating. It is calculated using a conformal transformation method and a linear approximation of the refractive index^31^. Each grating element only couples out the part of the WGM which is located exactly below the slit. Since they are phase shifted with respect to each other, annihilation between the outcoupled light from the red and blue areas in the WGM is expected. In case of equal slit sizes at 90° and 270° (see Fig. 3(a)) no complete annihilation occurs but a domination of the outer component. This is due to the asymmetric shape of the WGM. Maximum light annihilation occurs where the dividing line between red and blue grating coincides with the center of mass (COM) of the WGM, hence the red and blue areas of the WGM are equal. These points are symbolized in Fig. 3(a) by the two black circles. Compared to abrupt *π*-shifts^28^ the axis through both circles is rotated by a certain angle from the horizontal axis. This induces an overall rotation of the far field pattern. However, since the areas of strongest light emission stay at 0° and 180°, the tilt of the far field is not equal to the rotation of the points of strongest light annihilation. A proper determination of the far field rotation requires a detailed analysis of the spatial dependent near field amplitude. For this purpose the electric near field amplitude along the ring is calculated by the difference between the red and blue areas under the WGM curve. In addition, the reduced etch depth of small grating slits due to aspect ratio dependent etching^32^ is considered. As shown in Fig. 3(b) a decreased etch depth comes along with a reduction of the surface grating losses *α*_surf_. We assume constant total losses *α*_tot_ over the whole ring and find a linear dependence of the output power *P* = *α*_surf_/*α*_tot_ on the surface losses. The tangential electric field amplitude along the ring consequentially amounts to
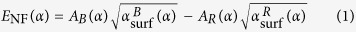
where 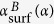 and 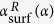 describe the surface losses of the blue and red slits, respectively. *A*_*B*_(*α*) and *A*_*R*_(*α*) denote the corresponding blue and red areas under the WGM curve. The normalized near field amplitudes for the horizontal and vertical polarization components are given by *E*_NF_(*α*)cos(*α*) and *E*_NF_(*α*)sin(*α*), respectively. They are depicted in Fig. 3(c). The final far field, containing both polarizations, is simply the sum of the horizontal and vertical far fields, each given by the squared 2D-Fourier transform of the corresponding near field amplitude. Experimental near fields of an abrupt and a continuous *π*-phase shift ring QCL are shown in Fig. 3(d,e), respectively. While the former shows a sudden intensity drop directly at the phase shifts, the latter exhibits a continuously decreasing near field intensity pattern towards the points of strongest light annihilation. See Supplementary information for a video showing a sketch of the near field and the calculated far field for the transition from abrupt to continuous *π*-phase shift.

DFB gratings incorporating a continuous *π*-phase shift were already discussed for straight waveguides in the form of a linear chirp of the grating^33^. In contrast to such a varying grating periodicity our dual DFB grating exhibits a constant grating periodicity but a varying ratio between *π*- and unshifted grating elements. Consequently, the near field amplitude resembles the amplitude of a chirped grating^27^. This pseudo-chirp modifies the near field amplitude as well as the far field pattern. For straight DFB lasers the linear grating chirp increases the near field intensity profile at the center of the laser and thus directs the optical power into one of the two main far field lobes producing an off-center single-lobed beam^33^. Compared to that, our dual DFB grating locally decreases the near field intensity profile and replaces the central intensity minimum by a central maximum. Reversing the direction of the grating chirp for a straight DFB laser would shift the intensity in the other far field lobe. For continuous phase shift ring lasers this would only change the WGM-induced rotation direction of the far field.

### Counter clockwise far field rotation - 1st order mode

**Figure 4:**
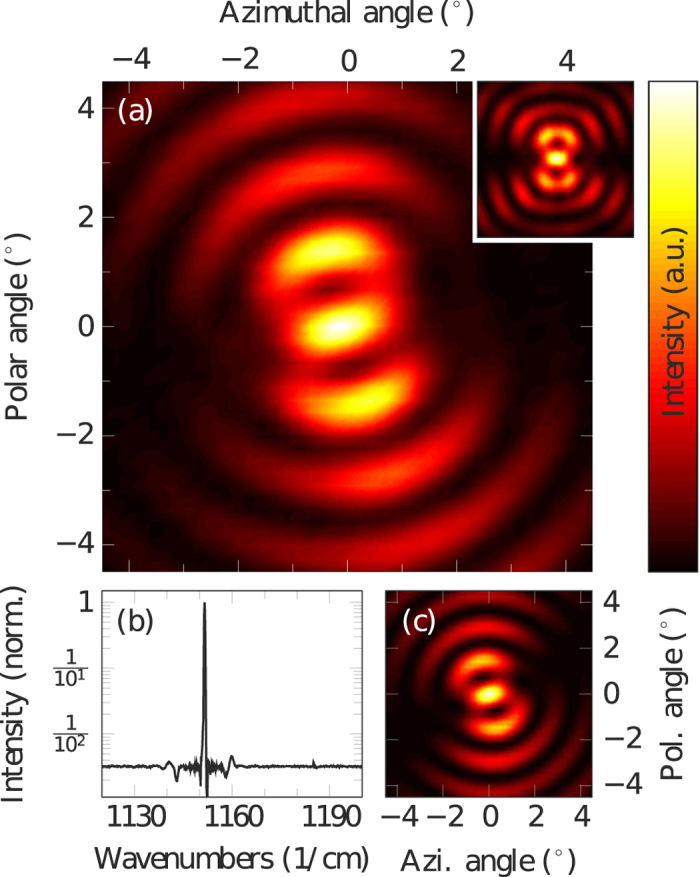
(**a**) Measured far field of a continuous *π*-shift laser with a counter clockwise rotation of the far field pattern. The inset shows the far field of an abrupt *π*-shift laser, which was used for alignment. (**b**) Single-mode spectrum of the continuous *π*-shift laser with a side mode suppression ratio of more than 23 dB. (**c**) Calculated far field.

**Figure 5:**
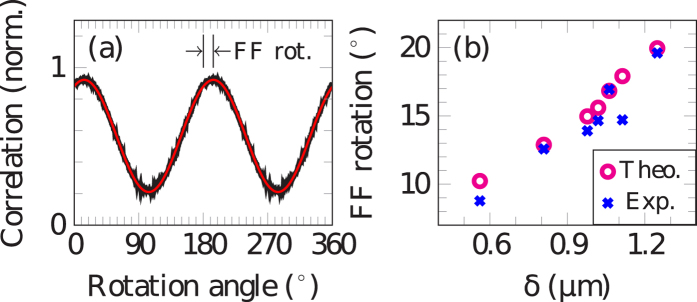
(**a**) 2D correlation coefficient of the revolving experimental far field from Fig. 4(a) and the reference far field. The sinusoidal fit (red) determines the far field rotation (FF rot.). (**b**) Experimental and theoretical far field rotation versus *δ*. A linear dependency with an inverse slope of (67 ± 5)nm/° for the experimental and (62 ± 4)nm/° for the theoretical data is found.

Figure 4(a) shows the measured far field of a ring QCL equipped with two continuous *π*-phase shifts in the DFB grating. The inset displays the far field of an abrupt *π*-shift ring QCL, fabricated on the same chip. This laser was used for alignment purposes. For our measured devices the overall output power of continuous and abrupt *π*-phase shifts was similar. Due to the narrower far field pattern of the continuous phase shift laser, the fraction of light in the central lobe over the entire measured far field is 9.8%, which is slightly higher than for the abrupt phase shift device with 8.5%. In addition, the polarization purity for the continuous phase shift lasers is higher. We define this value for the entire far field as the ratio between the polarization component 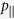 of the central lobe and the component *p*_⊥_ perpendicular to that. For the measured continuous and abrupt phase shifts this ratio is 1.67 dB and −0.97 dB, respectively. The minus sign implies that 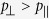. Both far fields show a central intensity maximum but while the far field’s symmetry axis of the abrupt *π*-shift is vertical, it is rotated counter clockwise for the continuous *π*-shift. The single mode spectrum of this laser with a side mode suppression ratio of more than 23 dB is presented in Fig. 4(b). The calculated far field is given in Fig. 4(c). In order to quantify the rotation of the far field, we used a calculated reference far field, where *E*_NF_ is a linear function (see Fig. 3(c), dashed black). This would describe the case in which the WGM is symmetrically centered in the waveguide and therefore the far field exhibits no tilt. While the experimental far field is rotated around the center point, the 2D correlation coefficient of this revolving far field and the reference far field is calculated for each angle. Its curve progression is shown in Fig. 5(a). The shift of the sinusoidal fit (red) reveals the rotation of the experimental far field, which amounts to 13.9°. In the same way the rotation of the theoretical far field is found to be 15.0°. This analysis was carried out for seven lasers with different waveguide and grating widths. Details are given in Table 1. The results are shown in Fig. 5(b). Rotation angles vary from 8.8° to 19.6°, depending on the distance
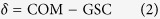
between the COM of the WGM and the grating slit center (GSC). In general, the larger *δ* the greater the rotation of the far field. From our experimental data we can extract an inverse slope of (67 ± 5)nm/°. This means that there is a direct correlation between the far field rotation and the location of the WGM relative to the grating. Altogether we observe a good agreement between the theoretical and experimental rotation of the far fields. A small initial tilt of the entire chip could be the reason for the fact that almost all far fields show a slightly smaller rotation compared to the theoretical prediction. Five of the seven measured ring lasers exhibit an error less than 1.1°. Possible error sources are processing imperfections like non-uniform etch depths and lift-off residuals on the grating slits, which influence the light outcoupling.

### Clockwise far field rotation - 2nd order mode

**Figure 6:**
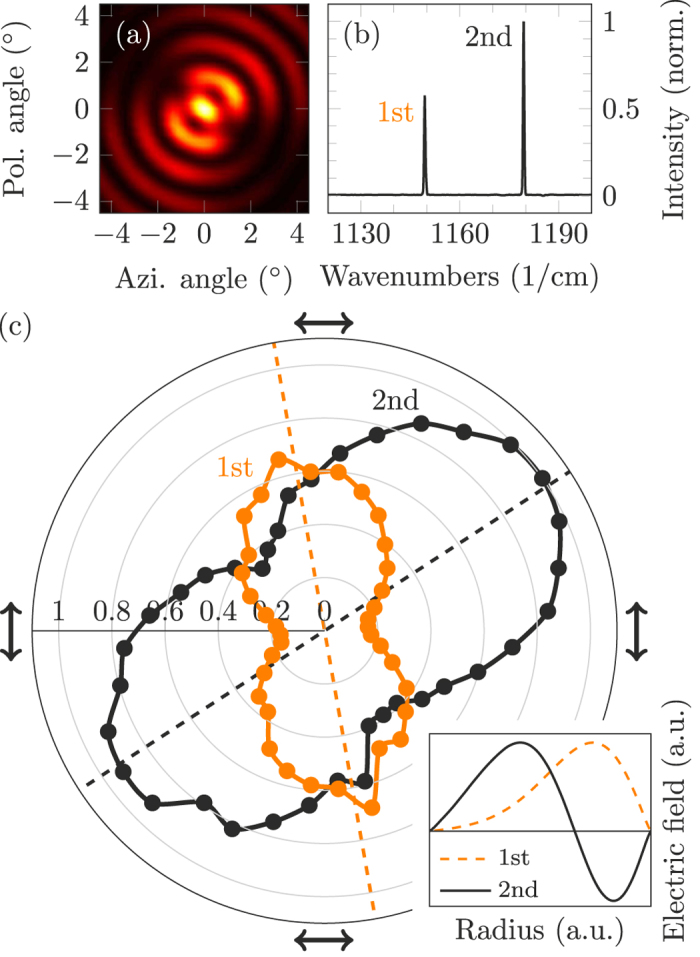
(**a**) Measured far field exhibiting a clockwise rotation. (**b**) Spectrum of this laser with two distinctive peaks. Their separation of roughly 30/cm agrees with our expectations considering the effective refractive index slope within the waveguide and the different locations of the modal COM. (**c**) Polar map describing the polarization dependent spectral peak heights from (**b**). The arrows denote the transmitted polarization and the dashed lines the calculated rotation of the symmetry axes. The inset shows the 1st order WGM and the different polarities of the 2nd order WGM, which are responsible for the clockwise rotation in (**a**).

Some of the recorded far fields from the same chip reveal a clockwise rotation of the far field pattern as shown in Fig. 6(a). This is counter intuitive since they emit the most light from a region of the ring, where the dual grating should provide strong light annihilation. As displayed in Fig. 6(b), the spectrum exhibits two peaks at 1150/cm and 1180/cm. Polarization dependent measurements of this spectrum were conducted. Figure 6(c) depicts the maximum intensity of each peak for different orientations of the polarizer. Assuming tangential polarization^21^,^28^, the emission pattern of the first peak is rotated counter clockwise and the pattern for the second peak is rotated clockwise. This implies that the peak at 1150/cm is emitted by a 1st order WGM and the other peak at 1180/cm is emitted by a 2nd order WGM. The inset demonstrates the key aspect, which is the different polarity of the two lobes of a 2nd order WGM. When the dividing line between the phase shifted grating elements coincides with the zero point of the 2nd order WGM, the different polarities in the two lobes cancel out the grating *π*-shift and provide maximum light outcoupling instead of destructive interference. The far field in Fig. 6(a) is the overlap of the far fields emitted by the 1st and 2nd order WGM. Since the latter shows a stronger emission peak in the spectrum, it dominates the far field.

## Conclusion

In summary, we present ring lasers equipped with a dual DFB grating forming two continuous *π*-phase shifts. The nature of the WGM in combination with the characteristics of this grating provide a rotation of the far field pattern. A direct correlation between this rotation and the location of the WGM relative to the grating is discovered. We developed a theoretical tool in order to predict this tilt and find good agreement between theory and experiment. Finally, we find that it is possible to draw conclusions about the order of the WGM from the direction of the far field rotation.

## Methods

### Semiconductor laser

The laser presented here is based on an In_0.52_Al_0.48_As/In_0.53_Ga_0.47_As active region^10^ on an InP substrate. It is emitting around 8.7*μ*m. In the first processing step the second order DFB grating is defined by electron beam lithography. Hereafter, a Ti/Au/Ni metalization is evaporated, which acts as an etch mask for the subsequent etching of the grating and the waveguide. During the waveguide etching, the grating slits are protected by SiN. The last steps include the isolation between neighboring devices and the deposition of the electrical contacts.

### Measurements

The near field measurements were performed with an *f* = 3.8 cm ZnSe lens and a microbolometer camera. All far field measurements were conducted at room temperature in pulsed mode with a repetition rate of 5 kHz and a pulse length of 100 ns. The far fields were recorded with a liquid nitrogen cooled mercury-cadmium-telluride detector with a pixel size of 1 mm^2^ mounted on a two-dimensional linear translational stage at a distance of 15 cm without any lenses in the beam path. A transformation of the far field was carried out to create a spherical far field pattern and correct for the angle dependent projection area at the detector. Spectral measurements were performed with a Fourier transform infrared spectrometer using a pulse length of 30 ns and a repetition rate of 80 kHz in order to reduce peak broadening due to intra pulse heating of the structure.

### Analysis

The aspect ratio dependent etching parameters were determined by focused ion beam milling of the grating structure. The grating-depth dependent surface losses *α*_surf_ as well as the total losses *α*_tot_ were calculated for an infinitely long straight DFB waveguide using coupled-mode theory^34^ based on coupling coefficients from on-resonant Floquet-Bloch solutions of the waveguide problem.

## Additional Information

**How to cite this article**: Szedlak, R. *et al*. The influence of whispering gallery modes on the far field of ring lasers. *Sci. Rep.* **5**, 16668; doi: 10.1038/srep16668 (2015).

## Supplementary Material

Supplementary Information

Supplementary Movie

## Figures and Tables

**Table 1:** Geometrical laser parameters (μm).

**W**_***i***_	**W**_***o***_	**G**_***w***_	**GSC**	**COM**	***δ***
189	201	4.4	194.90	195.46	0.56
189	201	5.2	194.68	195.49	0.81
189	202	5.6	195.22	196.20	0.98
189	202	5.6	195.09	196.11	1.02
189	202	5.7	194.95	196.01	1.06
189	203	5.4	195.23	196.34	1.11
189	203	5.6	194.63	195.88	1.25

Inner and outer waveguide radii are given by W_*i*_ and W_*o*_, respectively. G_*w*_ is the grating width and *δ* is the distance between the center of mass (COM) and the grating slit center (GSC).
