# Supplementary material for: The influence of whispering gallery modes on the far field of ring lasers
Source: Sci Rep. 2015 Nov 17;5:16668. doi: 10.1038/srep16668 (PMC4647838; doi:10.1038/srep16668)
Supplement: Supplementary Information [file srep16668-s1.pdf]

The influence of whispering gallery modes on the far field of ring lasers

Rolf Szedlak[1,*], Martin Holzbauer[2], Donald MacFarland[1], Tobias Zederbauer[2], Hermann Detz[2], Aaron Maxwell Andrews[1], Clemens Schwarzer[1], Werner Schrenk[2], and Gottfried Strasser[1,2]

[1]Institute of Solid State Electronics, TU Wien, Floragasse 7, 1040 Vienna, Austria
[2]Center for Micro- and Nanostructures, TU Wien, Floragasse 7, 1040 Vienna, Austria
[*]Corresponding author: rolf.szedlak@tuwien.ac.at

Supplementary Information

The video shows a sketch of the near field phase (top) as well as the corresponding calculated far field (bottom) for the transition from abrupt to continuous π-phase shift. It starts with an abrupt π-phase shift, where the upper part (red) of the ring is π-shifted and the lower part (blue) is unshifted. The calculated far field shows a central intensity maximum and a vertical symmetry axis. As the video proceeds, the near field phase is transformed into two continuous π-phase shifts. Consequently, the calculated far field exhibits a counter clockwise rotation of its symmetry axis.
